# Growth of *Spirulina* spp. at different temperatures and their impact on pigment production, oxidants and antioxidants profile

**DOI:** 10.1371/journal.pone.0313350

**Published:** 2025-02-24

**Authors:** Maha Rehman, Talha Shah, Talha Shabir, Iqra, Taj Ud Din, Mamata Chahar, Rajni Verma, Damanjeet Aulakh, Muhammad Daud, Rehan Naeem, Abdela Befa Kinki

**Affiliations:** 1 Department of Biotechnology and Genetic Engineering, Kohat University of Science and Technology, Kohat, Pakistan; 2 Department of Chemistry, NIMS Institute of Engineering and Technology, NIMS University Rajasthan, Jaipur, India; 3 Department of Applied Sciences, Chandigarh Engineering College, Chandigarh Group of Colleges-Jhanjeri, Mohali, Punjab, India; 4 Chitkara University Institute of Engineering and Technology, Centre for Research Impact & Outcome, Chitkara University, Rajpura, Punjab, India; 5 Department of Food Science and Nutrition, Ethiopian Institute of Agricultural Research Ethiopia, Addis Ababa, Ethiopia; Universiti Malaysia Kelantan, MALAYSIA

## Abstract

The study investigated the effect of different temperatures on the growth, pigment production, oxidative stress markers, and antioxidant profile of *Spirulina* spp. The results showed that the maximum growth of *Spirulina* was observed at 20°C, while the chlorophyll a, chlorophyll b, and carotenoid contents decreased at higher temperatures. Additionally, the concentration of oxidants such as MDA and H2O2 increased with temperature, while the concentration of antioxidants such as SOD, POD, and APX also increased with temperature. However, the optimal concentration of antioxidants varied with temperature, with the highest concentration of SOD and POD observed at 30°C and the highest concentration of CAT and APX observed at 20°C. Overall, the study suggests that the growth of *Spirulina* is temperature-dependent, with optimal growth observed at 20°C. Additionally, the study highlights the complex relationship between temperature and oxidative stress in *Spirulina*, with both oxidants and antioxidants increasing with temperature. However, the optimal concentration of antioxidants varies with temperature, indicating the need for further research to understand the mechanisms underlying these observations. The findings of this study have implications for the cultivation of *Spirulina*, particularly in regions where temperatures fluctuate significantly, and may also have implications for the use of *Spirulina* as a dietary supplement.

## 1. Introduction

Algae have a great contribution to biotechnology and are used in different domains including chemical, food, and cosmetic industries, biofuel production, and medicines [1]. These cellular factories are rich sources of natural products including core biomolecules and their derivatives [2,3]. They are photosynthesising organisms that alter light energy into chemical energy for their food production and have a simple reproduction mechanism [4]. Algae are classified into three types based on their biotechnological properties: cyanobacteria, microalgae, and macroalgae Cyanobacteria are sometimes classified as microalgae. The furthermost classification of microalgae encompasses both eukaryotic as well as prokaryotic organisms and creatures with one or more cells. Microscopic algae include microalgae, and prokaryotic Cyanobacteria [5]. The microalgal groups of major important characters are chlorophytes, bacillariophytes, while macroalgae are living in natural habitats [6]. Algae that are currently involved in different natural product formations including protein contents known as biotechnological cell factories is the cyanobacterium species *Arthrospira*, generally acknowledged as *Spirulina* [7]. Tracing back the history to approximately 3.6 billion ages ago, the *Spirulina* is Earth’s first-born plant which produces oxygen and has a basic role in the life forming of other organisms.

Algae has also nutritional values and produces the most nutritious, concentrated food, and provides potential benefits including antioxidants, phytonutrients, probiotics, and nutraceuticals [8]. It has a great role in health industries by producing varied demanding products which can be used as therapeutic agents [9].

Moreover, a lot of species have been discovered to be involved in the production of antioxidants and their derivatives which are attributes of normal healthy life [9]. Antioxidants are natural products that have the potential of retarding the oxidation of an already oxidizable chemical, which has to play a relevant role in cellular life, thus defending the cell against the negative action of oxidizing free radicals [10]. The compounds having phenolic properties which include phenols, phenolic acids, flavonoids, tannins, and lignins are scavengers for free radicals and hunt them with their redox properties [11]. The antioxidation activity of *Spirulina* has recently been reported and attracted much attention as in many functional studies it has shown a potential role in reducing oxidative stress [12]. It has been reported that these algae are nourished with many proteins, different carbohydrates, and a source of vitamins, carotenoids, and micronutrients, which can not only involve in the nutrition enhancements of animals but also improve the health aspects of animals and help in polishing the body color of the animal [8,13,14].

Commercial-scale algal cultivating in different conditions has been practiced for over a decade [15], mostly to yield natural high-protein supplements and other nutrients. The elevated expenses involved in cultivating algae have posed a significant obstacle to the commercialization efforts, as these organisms have a variety of growth conditions including nutrient availability, temperature maintenance, and light [16]. This core requirement has a great role in the development of *Spirulina* and its biomass arrangement created by triggering changes in their metabolism that significantly adjust the period of the assemblage of the main components of biomass [17]. The optimum temperature for the fruitful increase of *Spirulina* growth is approximately 18°C, while the culture growing below 12°C is not recommended suitable for the growth of *Spirulina*. The goal of our study was to regulate the influence of different temperatures on the growth profile of *Spirulina* in addition to analyze the photo pigmentation, antioxidative and oxidative profile of *Spirulina* at different temperatures.

## 2. Methods

### 2.1. Sample collection

**Fig 1:**
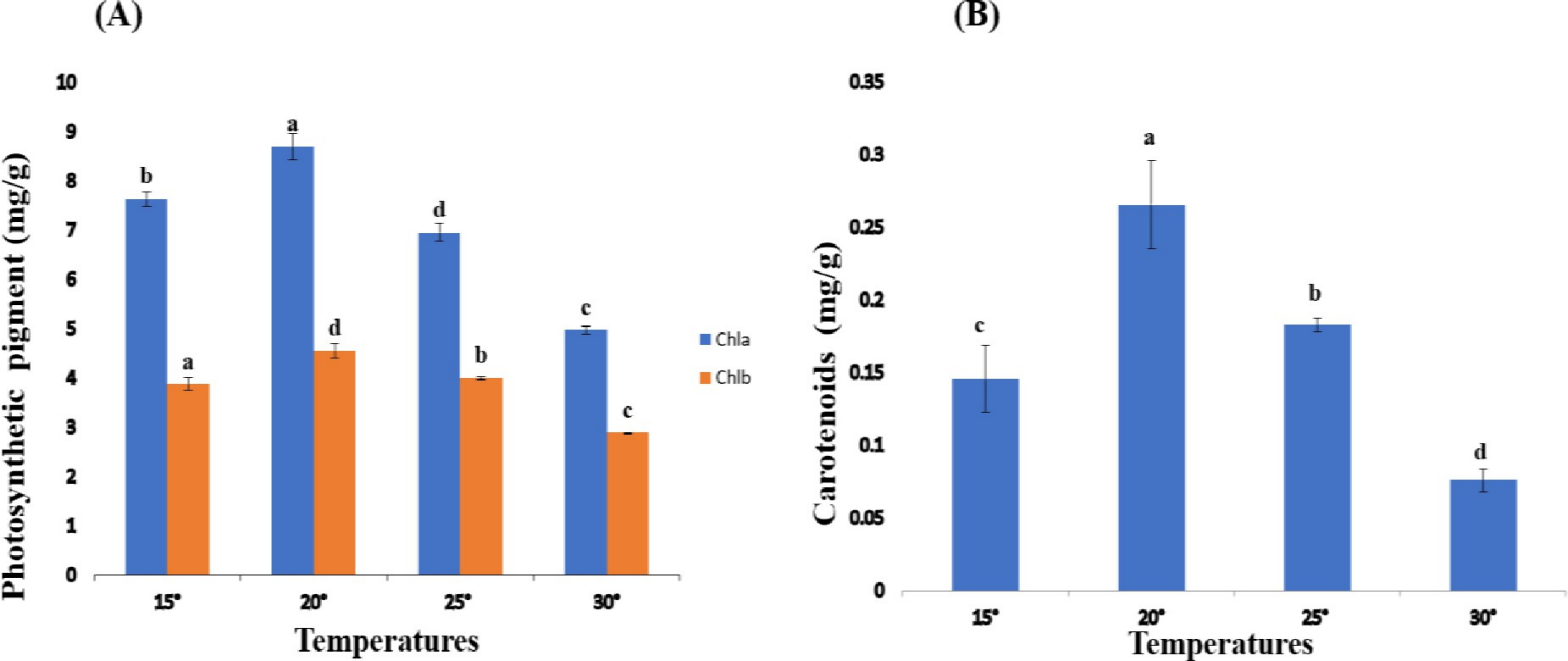
Microscopic view of *spirulina* spp.

Culture of *Spirulina* (*Arthrospira platensis*, accession number MT426015.1) was procured from the Molecular Ecology and Conservation Laboratory of Kohat University of Science and Technology, Kohat, Pakistan **(Fig 1)**. The culture was incubated in a shaking incubator specifically designed for the growth of *Spirulina* through which the ideal conditions including temperature was optimized. The research has been reviewed and approved by the Ethical Committee of Kohat University of Science and Technology (KUST), under the reference number KUST/Ethical Committee/463.

### 2.2. Growth analysis of *Spirulina* culture at different temperatures

*Spirulina* cultures were maintained in incubators at variable temperatures of 15°C, 20°C, 25°C, and 30°C, respectively, for a duration of 14 days. Temperature stability was ensured throughout the experiment by using thermostatically controlled incubators, which maintained constant temperatures with minimal fluctuations. The BG 11 medium was used to culture *Spirulina*, with the pH adjusted to 10.0 using hydrochloric acid (HCl) and sodium hydroxide (NaOH). The cultures were grown in a growth chamber with constant shaking at a speed of 20 rpm throughout the experiment to ensure uniform mixing and prevent sedimentation. They were exposed to a light intensity of 32.5 μmol m^2^ s⁻^1^ provided by 40-watt daylight fluorescent tubes that simulated daylight conditions. The optical density (OD) of the cultures at a wavelength of 450 nm was measured every 24 hours using a UV-Vis spectrophotometer [18].

### 2.3. Photopigment analysis

The analysis of photosynthetic pigments followed the modified methodology outlined by Eismann *et al*. (2020) [19]. The concentrations of chlorophyll a, chlorophyll b, and carotene were determined using the following formulas:

$$C_{a}=(15.65\times A_{666})-(7.340\times A_{65})$$ (i)

$$C_{b}=(27.05\times A_{653})-(11.21\times A_{666})$$ (ii)

$$C_{x+c}=[\left(1000\times A_{470})-(2.860\times C_{a})-(129.2\times C_{b}\right)]/245$$ (iii)

### 2.4. Determination of oxidative and antioxidants

Fresh biomass of *Spirulina* about 500 mg was used for the determination of oxidative stress markers and antioxidants from each treatment. *Spirulina* biomass was grounded in a 10 ml pre-cooled phosphate buffer containing (Na_2_HPO_4_.2H_2_O 16.385g\L and NaH_2_PO_4._ 2H_2_O 0.6663g\L). Then centrifugation was done at 20K rpm for a period of 20 min at 4°. The oxidative stress markers and antioxidant enzymes studied are listed below.:

#### 2.4.1. Oxidative stress markers

*2*.*4*.*1*.*1*. *Malondialdehyde*. The MDA content was determined by mixing 25 mg trichloroacetic acid (TCA), 2.5 mg thiobarbituric acid (TBA), and distilled water to a total volume of 500 ml. An enzyme extract was added to a reaction substrate, followed by incubation and subsequent centrifugation. Absorbance measurements at 532 nm and 600 nm allowed estimation of MDA-TBA levels, which were expressed as MDA g-1 fresh mass [20].

*2*.*4*.*1*.*2*. *Hydrogen peroxide (H*_*2*_*O*_*2*_*)*_`_The protocol of Nankano and Asada (1981) was used to determine hydrogen peroxide (H2O2). A 3 ml reaction mixture was created by combining 1.0 ml of potassium phosphate buffer (PBS), 2.0 ml of potassium iodide (KI), and 1.0 ml of enzyme extract. At 390 nm, absorbance was measured.

#### 2.4.2. Antioxidant analysis

*2*.*4*.*2*.*1*. *Super oxide dismutase (SOD)*. SOD content was analyzed using the Blanchamp and Fridovich (1971) method, involving the preparation of a substrate mixture and a reaction mixture containing enzyme extract, H_2_O_2_, and the substrate. The reaction mixture was exposed to light conditions for 20 minutes at 4000 lux, with light and dark controls provided. The reaction was stopped by turning off the lights, and the NBT photo reduction was conducted at 560 nm.

*2*.*4*.*2*.*2*. *Peroxidase (POD) activity*. Around 0.1 ml *Spirulina* enzyme extract, 0.1 ml of 1.5% guaiacol, 2.7 ml potassium phosphate buffer (PBS), and 0.1 ml 0.4% H_2_O_2_ were mixed to prepare 3 ml of the reaction mixture. The mixture was placed for 2 min and absorbance was recorded at 470 nm. A blank using 0.1 ml distilled water substituted the enzyme extract [21].

*2*.*4*.*2*.*3*. *Catalase (CAT)*. Catalase content was determined by following the protocol of [22]. A 3 ml of reaction mixture was prepared that contain 25 mM (2.8 ml) potassium phosphate buffer, 100 μl enzyme extract, and 30 Mm (100 μl) H_2_O_2,_ and absorbance was determined at 240 nm [22].

*2*.*4*.*2*.*4*. *Ascorbate peroxidase (APX)*. A reaction mixture was created, comprising 100 mM phosphate buffer (pH 7.0), 0.1 mM sodium EDTA, 0.3 mM ascorbic acid, 0.06 mM H2O2, and 100 μL of enzyme extract. The absorbance of the mixture was recorded at a wavelength of 290 nm [23].

### 2.5. Statistical analysis

The analysis of variance (ANOVA) and standard deviation (SD) of the obtained data were performed using Statistix 9.0 software.

## 3. Results

### 3.1. Growth at different temperatures

**Fig 2:**
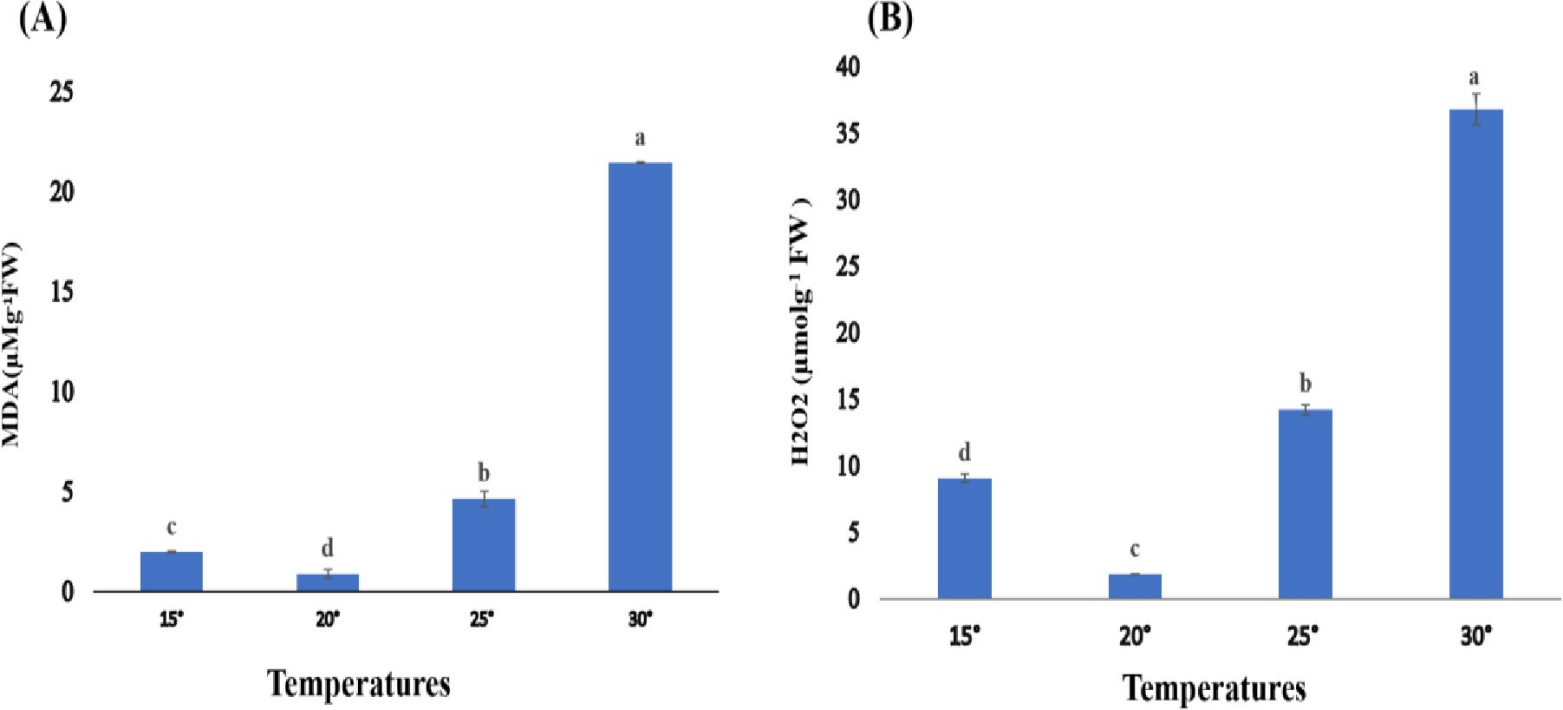
Growth of *Spirulina* spp. at 450nm for different temperature. Values are expressed as the mean ± S.D, based on triplicate measurements. Error bars represent standard deviations, with significance established at p < 0.05. Distinct alphabetic letters denote statistically significant differences as determined by one-way ANOVA using Statistix 9.0 software.

*Spirulina* growth was evaluated at different temperatures for a period of 7 days. The growth of *Spirulina* biomass cultivated at variable temperatures has shown a significant change (P < 0.05) in biomass. The maximum concentration of biomass (0.84) was reported at a temperature of 20°C while biomass concentration (0.31) was found to be reduced at a temperature 30°C. A wide sequence of temperature tolerance was observed for the growth of *Spirulina* at variant temperatures extending from 15 to 30°C. But at 30°C, the growth was reported as minimum as shown in (**Fig 2)**.

### 3.2. Photo pigment production

#### 3.2.1. Analysis of chlorophyll a and chlorophyll b contents

**Fig 3:**
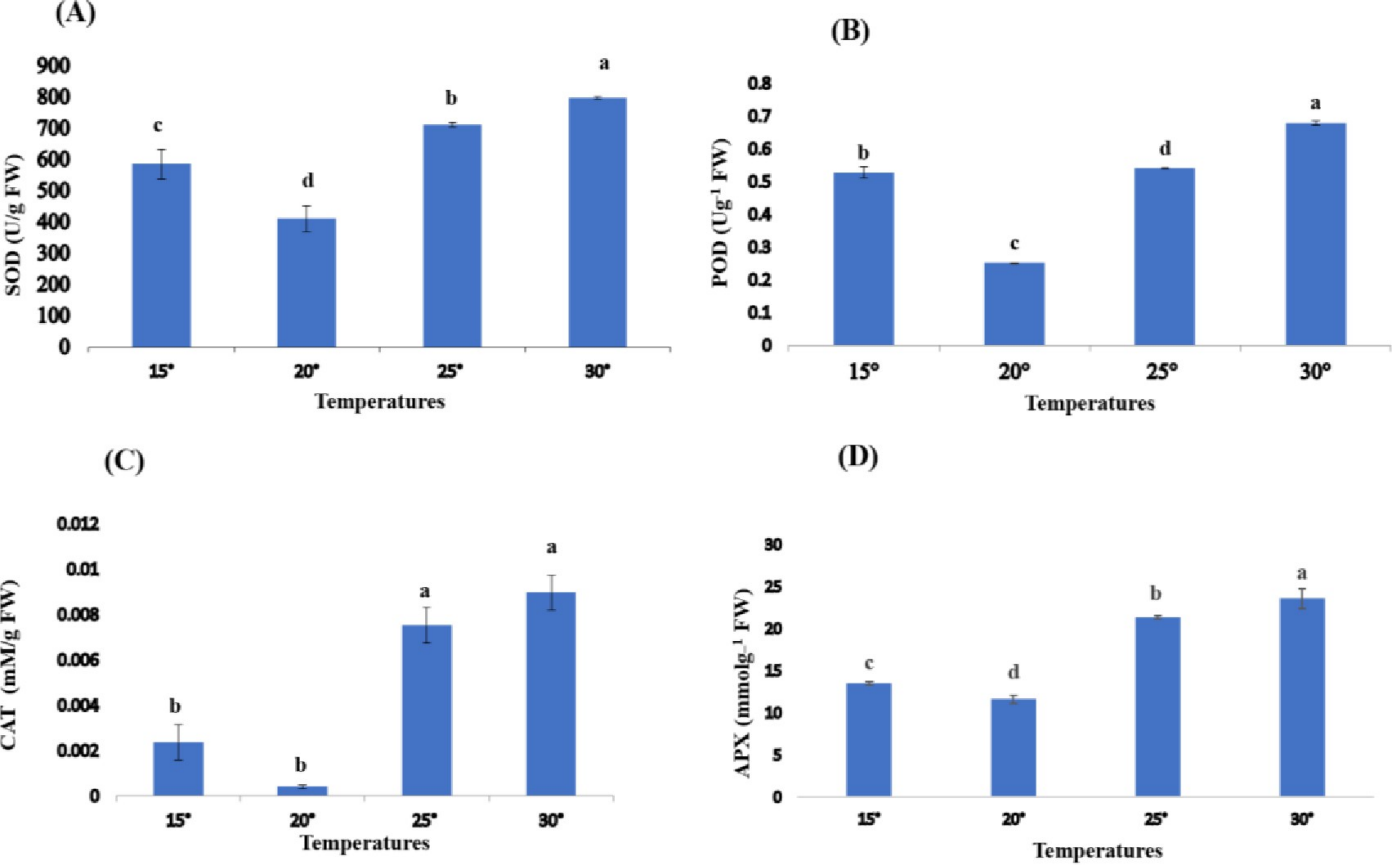
**(A, B).** Photosynthetic pigment concentration of *Spirulina* spp. at different temperature. Values are expressed as the mean ± S.D, based on triplicate measurements. Error bars represent standard deviations, with significance established at p < 0.05. Distinct alphabetic letters denote statistically significant differences as determined by one-way ANOVA using Statistix 9.0 software.

The whole content of chlorophyll a and chlorophyll b of *Spirulina* was evaluated at different temperatures for 7 days. The chlorophyll a and chlorophyll b content at variable temperatures have shown significant change (P< 0.05) in concentration. The maximum concentration of photo pigment; chlorophyll a (8.68 mg/g) and chlorophyll b (4.54 mg/g) were reported at 20°C for *Spirulina* spp. while photopigment concentration was found to reduced (chlorophyll a (4.96 mg/g), chlorophyll b (2.88 mg/g)) respectively at 30°C as shown in (**Fig 3A).**

#### 3.2.2. Carotenoids

Carotenoid content for *Spirulina* was evaluated at different temperatures for 7 days. Carotenoid content at different temperatures has shown a substantial change (P< 0.05) in the whole concentration. The maximum concentration of carotenoids (0.26 mg/g) was reported at 20°C while carotenoid concentration (0.075 mg/g) was found to be reduced at a temperature 30° C as shown in (Fig 3B).

### 3.3. Oxidative stress makers

#### 3.3.1. Malondialdehyde

**Fig 4:**
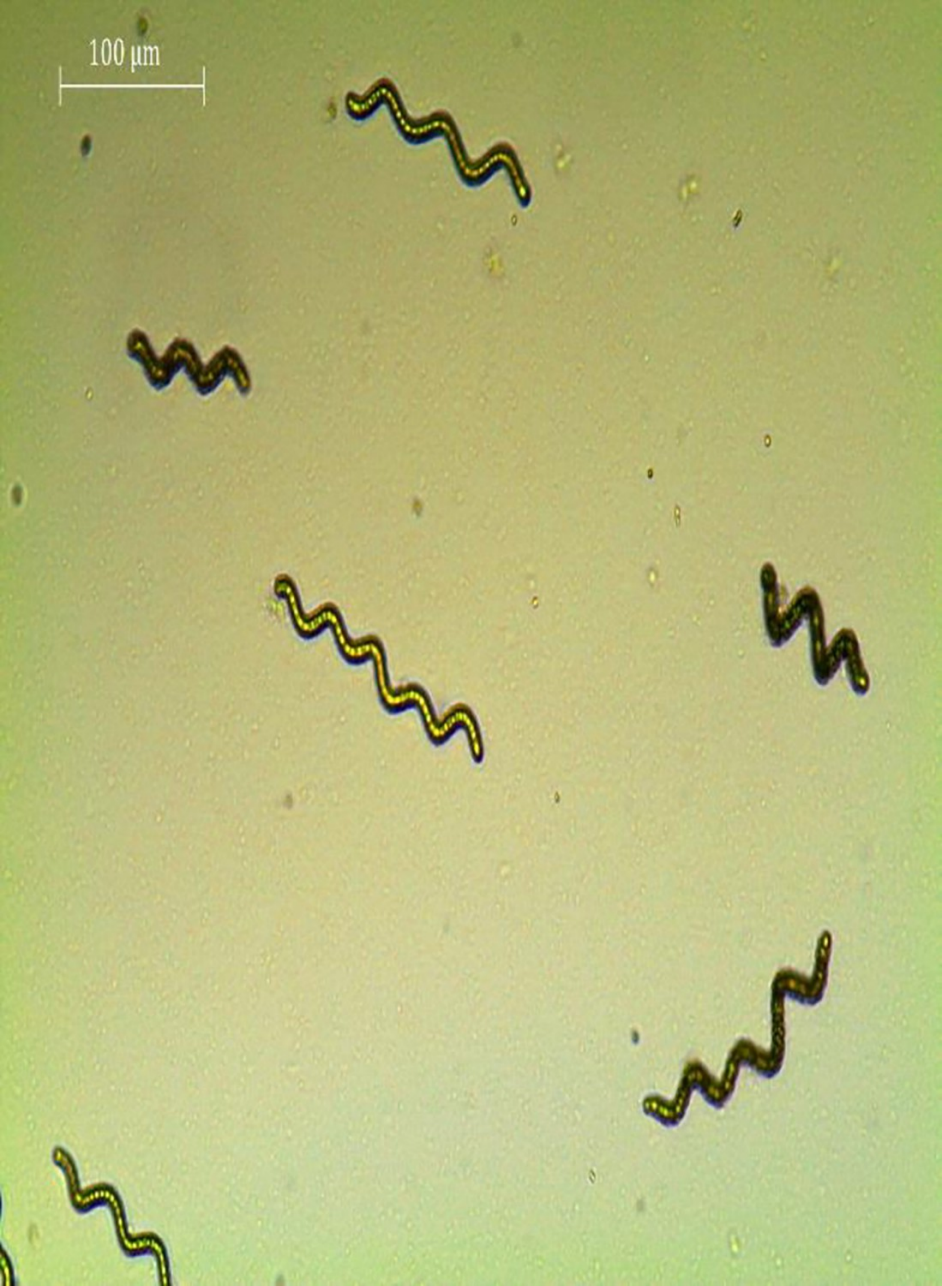
**(A, B)**. Malondialdehyde (MDA) Hydrogen peroxide (H_2_O_2_) as oxidative stress marker for *Spirulina* spp. at different temperature. Values are expressed as the mean ± S.D, based on triplicate measurements. Error bars represent standard deviations, with significance established at p < 0.05. Distinct alphabetic letters denote statistically significant differences as determined by one-way ANOVA using Statistix 9.0 software.

MDA content of *Spirulina* was evaluated at different temperatures for 7 days. MDA content at variable temperatures shown a significant change (P< 0.05) in whole concentration analysis. The maximum concentration of MDA (21.445 μM^-1^g FW) was reported at a temperature 30°C while MDA concentration (0.903 μM^-1^g FW) was found to be reduce at 20°C as shown in (Fig 4A).

#### 3.3.2. Hydrogen peroxide

The Hydrogen peroxide (H_2_O_2_) content of *Spirulina* was evaluated at different temperatures for 7 days. H_2_O_2_ content at variable temperatures shown a significant difference (P< 0.05) in concentration. The maximum concentration of H_2_O_2_ (36.76 μmolg^-1^ FW) was reported at a temperature of 30°C while H_2_O_2_ concentration (1.833 μmolg^-1^ FW) was found to be reduce at 20°C as shown in (Fig 4B).

### 3.4. Antioxidant determination

#### 3.4.1. Superoxide dismutase

**Fig 5:**
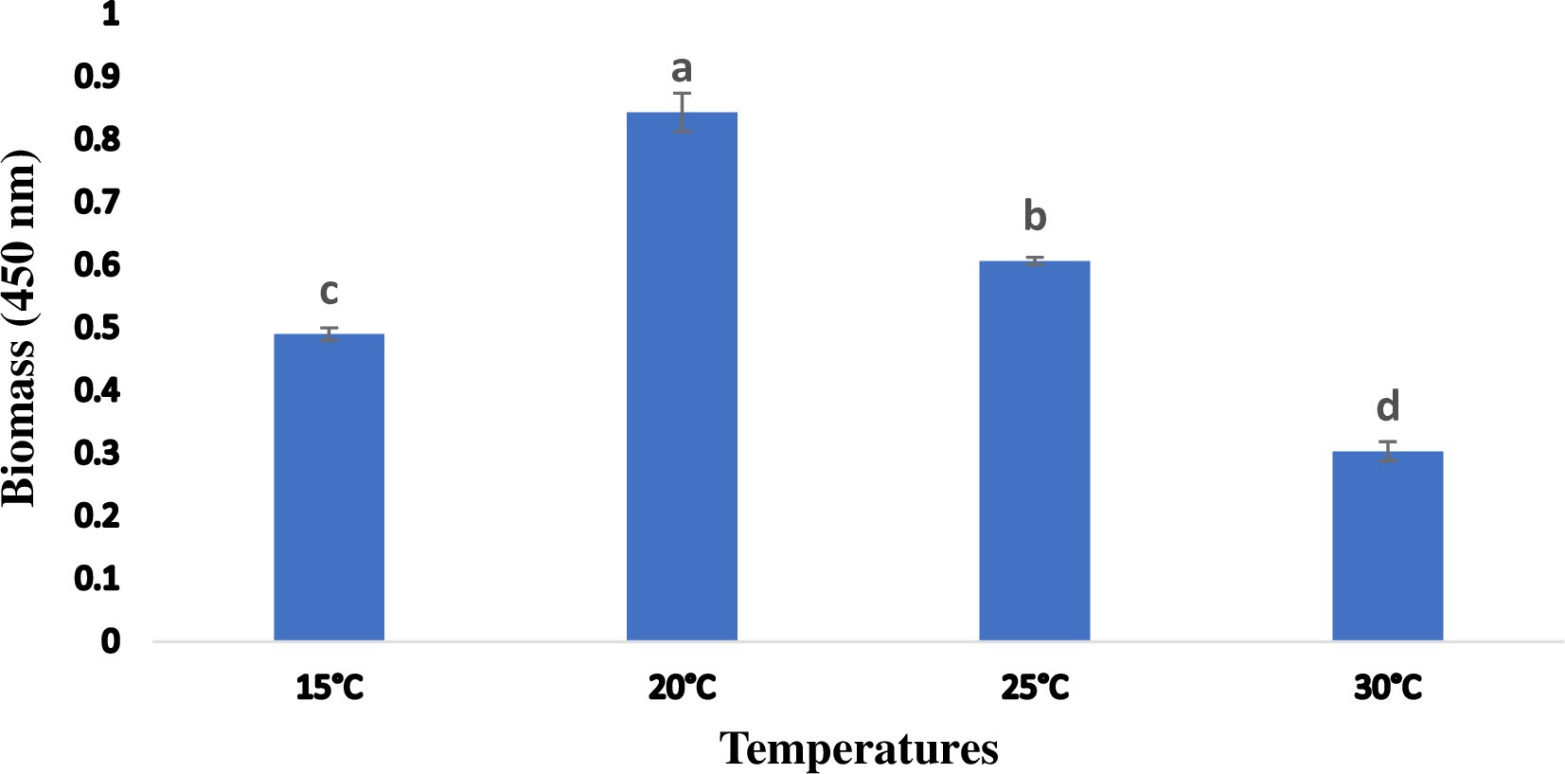
**(A, B, C and D)**. Antioxidant markers for *Spirulina* spp. at different temperature. Values are expressed as the mean ± S.D, based on triplicate measurements. Error bars represent standard deviations, with significance established at p < 0.05. Distinct alphabetic letters denote statistically significant differences as determined by one-way ANOVA using Statistix 9.0 software.

Antioxidant content including Superoxide Dismutase (SOD) content for *Spirulina* was evaluated at different temperatures for 7 days. SOD content at variable temperatures shown a significant difference (P< 0.05) in concentration. The maximum concentration of SOD (797.19 Ug^-1^ FW) was reported at a temperature of 30°C while SOD concentration (410.04 Ug^-1^ FW) was found to be reduce at 20° C as shown in (**Fig 5A).**

#### 3.4.2. Peroxidase

Peroxidase (POD) content of *Spirulina* was evaluated at different temperatures for 7 days. The POD content at temperatures showed a significant difference (P< 0.05) in concentration. The maximum concentration of POD (0.67 Ug^-1^ FW) was reported at a temperature 30°C while POD concentration (0.25 Ug^-1^ FW) was found to be reduced at 20°C as shown in (Fig 5B).

#### 3.4.3. Catalase

Catalase (CAT) content of *Spirulina* was evaluated at different temperatures for 7 days. CAT content at a variable temperature shown a significant difference i-e (P< 0.05) in concentration. The maximum concentration of CAT (0.009 mMg^_1^ FW) was reported at a temperature 30°C while CAT concentration (0.00041 mMg^_1^ FW) was found to be reduced at 20°C as shown in (**Fig 5C)**.

#### 3.4.4. Ascorbate peroxidase (APX)

APX content of *Spirulina* was evaluated at different temperatures for 7 days. APX content at variable temperatures shown a sustainable difference (P< 0.05) in concentration. The maximum concentration of APX (23.58 mmolg^_1^ FW) was reported at a temperature of 30°C while APX concentration (11.61 mmolg^_1^ FW) was found to reduce at 20° C as shown in (Fig 5D).

## 4. Discussion

Growth of *Spirulina* culture analysis was evaluated at different temperatures has shown variable growth profiles i-e at 15°C, 20°C, 25°C, and 30°C. Growth of *Spirulina* at 20°C was found maximum (0.85) followed by growth of *Spirulina* at 25°C (0.64), while at 15°C growth of *Spirulina* was lower (0.48). In the same way, the growth of *Spirulina* at 30°C was minimum (0.30). The graph of the growth pattern **(Fig 2)** of *Spirulina* cultures at various temperatures revealed that *Spirulina* has a temperature tolerance at 15°C, 20°C, and 25°C while the growth of *Spirulina* at 30°C was not exponential, revealing that 30° C was not optimal for the growth of *Spirulina* spp. Similar, results were also proposed by Vonshak *et*.*al*., (2003), they revealed that the minimum temperature, which was ideal for the growth of *Spirulina* was approximately 18°C while the culture growing below 12°C was not appropriate for the growth of *Spirulina*. It was found in the current study, that temperatures above 30°C were not optimal for the growth of *Spirulina*, while 20°C was the most optimal temperature for the growth of *Spirulina*, at which growth of *Spirulina* was severely affected. Monitoring of temperature is critical for the growth of *Spirulina* revealing that higher and lower temperature in comparison to optimal temperature has severely affected the growth of *Spirulina* and their cell structure components (lipids and proteins) along with their metabolism was affected [24,25]. Also, differences in temperature could be due to variations in *Spirulina* strains, cultivation conditions, or unique environmental factors in our study’s setting supported by the work of Soni *et al*. (2019) [6].

It was observed that Chlorophyll a, Chlorophyll b, and carotenoids contents of *Spirulina* spp. decreased with an increase in temperature. Similarly, Chlorophyll a (8.68 mg/g), Chlorophyll b (4.54 mg/g), and carotenoids (0.16 mg/g) were found maximum for *Spirulina* spp. cultures growing at 20°C. It was also evident that the Chlorophyll a (4.96 mg/g), Chlorophyll b (2.88 mg/g), and carotenoids (0.07 mg/g) were minimum at 30°C while these contents at 25°C were increased revealing 6.94 mg/g for Chlorophyll a, 3.99 mg/g for Chlorophyll b and 0.18 mg/g for carotenoids. Similarly, at 15°C, the Chlorophyll a (7.93 mg/g), Chlorophyll b (3.87 mg/g), and carotenoids (0.14 mg/g) were increased. Contrary results were also proposed by Guedes *et al*. (2011) [26], who specified that temperature theatres an important role in the growth and antioxidant content of the microalga *Scenedesmus obliquus*. The highest rate of biomass-specific growth and biomass productivity were associated with relatively low pH (6) and relatively high temperature (30°C). In another related study, Cheng *et al* (2016) [27] proposed that the growth, and content of chlorophyll *a* (*Chl a*), chlorophyll *b* (*Chl b*), and, carotenoids were progressively decreased with increasing the abiotic stress i-e Cadmium concentration, over 18 days of exposure. They noticed that chlorophylls (*Chl a* and *Chl b*) and carotenoids contents of *C*. *vulgaris* significantly declined with rising concentration of a-biotic stress (Cadmium concentration). It was earlier proposed by Chalanika De Silva and Asaeda (2017) that *chl a* and *chl b* for *E*. *nuttallii*, was significantly increased at 30°C while it was decreased at 35°C.

Temperature has a significant impact on microalgae CO2 capture and its utilization. At high temperatures, the concentration of MDA increased with an increase in temperature. In the same way, the concentration of MDA was found maximum (21.45 μM^-1^g FW) for *Spirulina* spp. cultures growing at 30°C. It was also evident that the concentration of MDA was minimum (0.90 μM^-1^g FW) at 20°C while MDA content at 15°C was 1.97 μM^-1^g FW and at 25°C, these contents were 4.64 μM^-1^g FW. Xing *et al*. (2022) showed that temperature stress caused in a significant increase in reactive oxygen species (ROS). It was deduced that algal strains were powerless to tolerate temperatures above 40°C, which indicates that the temperature series of 38 to 40°C is the upper limit for the growth of *Chlorella* strains [28]. Survival against oxidative stress is dependent on the balance of ROS creation and elimination by a variety of enzymatic and non-enzymatic detoxifying classifications. Earlier Cheng *et al*. (2016) [27] reported the same levels of hydrogen peroxide then superoxide anion, rising with increasing in the abiotic stress. It was noticed in the current study that at optimal temperature (20°C), the concentration of H_2_O_2_ was 1.83 μmolg^-1^ FW while at 30°C, the concentration of H_2_O_2_ was i μmolg^-1^ FW increased to 36.7 μmolg^-1^ FW with an increase in temperature. In the same way, the concentration of H_2_O_2_ was lower 9.06 μmolg^-1^ FW at 15°C while H_2_O_2_ content at 25°C was 14.2 μmolg^-1^ FW. Previously, it was suggested that exposing coleoptile cells to high temperatures during the later stages of seedling development leads to an enhanced production of O_2_^–^.

The increase in O_2_^–^ production, at all stages of development, led to an rise in MDA concentration [29]. Similarly, the effect of abiotic stress (Cd (II)) on hydrogen peroxide then superoxide anion was also analyzed. They noticed that the maximum concentration of ROS was observed in *C*. *vulgaris* cells treated with high concentrations of a-biotic stress (Cd (II)) in the 18 days of cultivation [27]. Reactive oxygen species (ROS) have the ability to directly harm proteins, amino acids, nucleic acids, and lipids present in cell membranes.

The antioxidants including SOD were found optimal (410.04 Ug^-1^ FW) for the growth of *Spirulina* at 20°C. while these were found higher (585.58 Ug^-1^ FW) at 15°C, similarly, their levels were increased to maximum 797.13 Ug^-1^ FW at 30°C followed by 711.25 Ug^-1^ FW at 25°C. In the same way, antioxidants including POD were found optimal 0.25 Ug^-1^ FW for the growth of *Spirulina* at 20°C. while these were higher 0.52 Ug^-1^ FW at 15°C. In the same way, their levels were increased to a maximum (0.67 Ug^-1^ FW) at 30°C followed by 0.54 Ug^-1^ FW at 25°C. CAT was also found optimal 0.0004 mMg^_1^ FW for the growth of *Spirulina* at 20°C. while these were higher (0.0089 mMg^_1^ FW) at 30°C followed by 0.0023 mMg^_1^ FW at 15°C and 0.0007 mMg^_1^ FW at 25°C. Similarly, APX was also found optimal 11.61 mmolg^_1^ FW for the growth of *Spirulina* at 20°C, while these were lower 13.51 mmolg^_1^ FW at 15°C. In the same way, these were a maximum 23.58 mmolg^_1^ FW at 30°C followed by 21.31 mmolg^_1^ FW at 25°C.

The protection systems of antioxidants such as superoxide dismutase (SOD), peroxidase (POD) also catalase (CAT), can eradicate the surplus ROS which are inducing stress factors for the cell, thus protect the cells against injury. Superoxide dismutase (SOD) is the first crucial enzyme to hijack the ROS in the cells. It has an important role in active oxygen eradication pathway, which can metabolize the O_2_^−^ to be H_2_O_2_, thereby has play a basic role in the protection of cells from oxidative free radical damage. POD is another enzyme found in the plants and act as an antioxidant enzyme defense system, its main activity is to promote the capacity of the antioxidants and to remove the severity of poisoning of plants, and is reported to catalyze the breakdown of the toxicity in a good series [30]. POD was reported to be involve in the reaction of H_2_O_2_ into H_2_O. CAT is also a great role in the removal of oxygen species and act as a ubiquitous enzyme as it removes H_2_O_2_ generated in the different metabolic pathways in the plant [26]. Guedes *et al*. (2011) proposed that the antioxidant production rate was increased with an increase in a-biotic stress including pH and temperature. To date, the highest productivity reported (0.638 mg L^−1^ day^−1^) at a pH of 8 at 30°C for microalga *Scenedesmus obliquus*. Previous findings indicated that the highest temperature corresponded to the highest antioxidant production. Additionally, it was suggested that temperature exerted a greater influence on the growth and productivity of algal species. Conversely, the rate of antioxidant production exhibited an upward trend as temperature increased [26].

### 4.1. Conclusions and recommendations

Growth of *Spirulina* biomass with high pigment contents was optimal at 20°C, while these were lower at 15°C, 25°C and 30° C. Oxidants including MDA, H_2_O_2,_ and antioxidants including CAT, SOD, POD, and APX were lower at optimal temperature (20°C) while these were higher at 15°C, 25°C, and 30°C, so it has be determined that growth of *Spirulina* was temperature dependent, and at 20°C, the growth of *Spirulina* was optimal at which the photosynthetic pigments were higher while oxidants and antioxidants were lower.

## Supporting information

S1 Data Supplementary data file of graphs. (XLSX)

S1 File (PDF)
