# Supplementary material for: Growth of Spirulina spp. at different temperatures and their impact on pigment production, oxidants and antioxidants profile
Source: PLoS One. 2025 Feb 24;20(2):e0313350. doi: 10.1371/journal.pone.0313350 (PMC11849831; doi:10.1371/journal.pone.0313350)
Supplement: S1 File — (PDF) [file pone.0313350.s002.pdf]

KOHAT UNIVERSITY OF SCIENCE & TECHNOLOGY

Kohat 26000, Khyber Pakhtunkhwa, Pakistan Ph # 0922-554563-554565/4786, 4785, Fax # 554556

Ref. No. /KUST/Ethical Committee/ 463
Dated: 07/09/2022

KUST Ethical Committee Approval Certificate

The Thesis/Synopsis: FOLIC ACID PRODUCTION OF SPIRULINA SPP. ISOLATED FROM WATER RESERVOIRS OF KOHAT, (PAKISTAN) AND ITS UTILIZATION AS FOOD SUPPLEMENTS

Submitted by: **MAHA REHMAN**

Registration No: **BT420201002**

Through: ____________________
(Chairman/Director)

After fulfilling the required documents, the KUST ethical Committee, hereby approved the above title.

7/9/2022

Chairman/Director, **KUST Ethical Committee**

Kohat University of Science & Technology
ACADEMIC
